# High burden of depression among cancer patients on chemotherapy in University of Gondar comprehensive hospital and Felege Hiwot referral hospital, Northwest Ethiopia

**DOI:** 10.1371/journal.pone.0237837

**Published:** 2020-08-21

**Authors:** Adhanom Gebreegziabher Baraki, Getahun Mengistu Tessema, Eyayaw Adisu Demeke

**Affiliations:** 1 Department of Epidemiology and Biostatistics, College of Medicine and Health Sciences, Institute of Public Health, University of Gondar, Gondar, Ethiopia; 2 Department of Internal Medicine, College of Medicine and Health Sciences, School of Medicine, University of Gondar, Gondar, Ethiopia; 3 Department of Physiotherapy, Bahirdar University, Bahirdar, Ethiopia; Ordu University, TURKEY

## Abstract

**Introduction:**

Cancer, the most stressful event a person may experience often triggers depression. Depression among these groups of people, in turn, affects chemotherapy adherence, length of hospitalization, quality of life and cancer treatment outcome. Even though the problem is enormous studies that address it are limited. Therefore this study was conducted to determine the prevalence of depression and associated factors among cancer patients on chemotherapy in Felege-Hiwot referral hospital and University of Gondar referral hospital, Northwest Ethiopia.

**Methods:**

An institution-based cross-sectional study was conducted from April to May 2019. A total of 302 cancer patients on chemotherapy were included. Depression was assessed using the patient health questionnaire (PHQ-9). Binary logistic regression was used to select variables and determine Crude Odds Ratio (COR). Variables with P value < 0.2 were entered into multivariable logistic regression. Adjusted Odds Ratio (AOR) with 95% confidence intervals for variables with P-value < 0.05 was estimated to show factors affecting depression among cancer patients. The fitness of the model was checked by using the Hosmer-Lemeshow goodness-of-fit test.

**Results:**

The prevalence of depression among cancer patients on chemotherapy was 70.86% (95% CI: 65.38, 75.92). Educational status of college and above (AOR = 0.1, 95% CI: 0.02, 0.43), Jobless (AOR = 0.15, 95% CI: 0.04, 0.58), Underweight(AOR = 2.39, 95% CI: 1.10, 5.19)chemotherapy duration ≥ 6 months or more (AOR = 2.36, 95% CI: 1.16, 4.79) were notably associated with depression.

**Conclusion:**

The burden of depression among cancer patients in this study was high. We recommend concerned bodies working to curve the problem to intervene based on the identified risk factors. Improving educational status, reducing work stress and maintaining normal weight would reduce depression.

## Introduction

The global burden of cancer has risen to 18.1 million new cases and 9.6 million deaths in 2018. Worldwide, the total number of people who are alive within 5 years of a cancer diagnosis is estimated to be 43.8 million [1].

Depression is a common mental disorder, characterized by persistent sadness and a loss of interest in activities that one normally enjoys, accompanied by an inability to carry out daily activities, for at least two weeks. More than 300 million people are now living with depression, an increase of more than 18% between 2005 and 2015 [2]. The national prevalence of depression among the general population in Ethiopia was 9.1% [3].

Cancer, the most stressful event that a person may experience often triggers depression [4, 5]. The prevalence of depression among cancer exceeds that observed in the general population [6] and it ranges from 16.4% to 66.72% [4, 7–12]. Depression among cancer patients affects treatment since they have to take medications for both cancer and depression [4], affect acceptance of adjuvant cancer treatment [13], adherence [14] extend hospitalization, reduces the quality of life [15, 16], and increases the risk of suicide [17]. Depression also predicts cancer progression and mortality [6, 18].

Several factors affect depression among cancer patients; these include age, sex, marital status, educational status, occupation, pain, type of cancer, phase of treatment [4, 8, 11], and social support [11, 19, 20].

Even though routine screening of distress is recommended internationally for good cancer care [21] less emphasis is given in the study area and most of the care focuses on cancer. Studies on the magnitude and the contributing factors are also limited. Therefore this study was conducted to fill this information gap by determining the prevalence of depression among cancer patients and factors affecting it.

## Methods

### Study design and period

An institution-based cross-sectional study was conducted among cancer patients from April to May 2019.

### Study area

This study was conducted on cancer patients who are getting treatment and have followed up at the oncology unit of the University of Gondar comprehensive specialized hospital (UoGCSH) and FelegeHiwot referral hospital (FHRH). The two hospitals are found in the Amhara region northwest Ethiopia 738 km and 565 km away from the capital Addis Ababa respectively. The oncology unit of UoGCSH currently has 10 beds for the management of cancer patients, whereas the oncology unit of FHRH has currently 18 beds for inpatient treatment of cancer patients.

### Participants

The source populations were all adult cancer patients visiting the oncology unit and treated with chemotherapy in these hospitals. All adults with any type of cancer patients under chemotherapy treatment and follow up during the study period were included in the study.

### Sample size and sampling procedure

A final sample size of 302 was found by using single population proportion formula with population correction for total cancer patients of 1400 in the two hospitals, using the following assumptions; the prevalence of depression = 50%, Z_a/2_ for 95% confidence interval = 1.96 and margin of error of 5%.

The final sample size was proportionally allocated to the two hospitals 126 for UoGCSH and 176FHRH. A systematic random sampling method was employed to select every 3^rd^ patients who were coming to the oncology unit during the data collection period and full fill the inclusion criteria. We had a plan to randomly select and replace the non-responders, but no study participant refused to participate.

### Variables

The dependent variable of depression was measured using the widely used Patient Health Questionnaire (PHQ-9). The Amharic (Local language) version of the scale has been validated in Ethiopia (sensitivity = 86% and specificity = 67%) [22]. We have used a cutoff point of 10 to classify patients as having depression or not.

Independent variables like Age, sex, marital status, average monthly income, educational level, smoking habit, alcoholic habit, physical activity were collected by interviewer-administered questionnaire whereas variables like Body Mass Index, Type of cancer, clinical-stage, type of chemotherapy, Duration of chemotherapy and, co-morbidities like Hypertension, DM, HIV, and Anemia were collected from patient charts. The smoking and Alcohol use habits were assessed by asking the patients if they ever smoke cigarette or drink alcohol, and social support was evaluated using the Oslo 3 item social support scale with scores ranging from 3 to 14 (poor = 3–8, moderate = 9–11, and strong = 12–14) [23].

### Data collection procedure and quality assurance

The data was collected by interviewing the participants using a structured pretested questionnaire and chart review. The data was collected by three nurses working in each oncology unit. Data collectors were trained for one day about the objective of the study and ethical considerations. Data collectors were supervised by the principal investigator. Data was reviewed and checked for completeness, accuracy, and consistency after each day of data collection.

### Data processing and analysis

Data were entered into Epi-info version 7.0 and STATA version 14 was used for analysis. Frequencies and percentages were computed for all variables. Data were presented in tables and graphs. Binary logistic regression was used to select variables and to determine Crude Odds Ratio (COR). Variables with P value < 0.2 were entered into multivariable logistic regression. Adjusted Odds Ratio (AOR) with 95% confidence intervals for variables with P-value < 0.05 was estimated to show factors affecting depression among cancer patients. The fitness of the model was checked by using the Hosmer-Lemeshow goodness-of-fit test.

### Ethics statement

Ethical approval to conduct the study was received from the University of Gondar ethical review board, School of Medicine (Reference number SOM/1237/2019). A permission letter was received from the two hospitals. To keep the privacy of participants’ name and other personal identifiers were not collected. Consent to participate in the study was also orally taken from patients. Patients with depression were also linked to the psychiatry clinic.

## Results

### Socio-demographic characteristic of study participants

A total of 302 patients participated in the study. From the total respondents, the majority of them 194(64.2%) were females, 221(73.2%) were married and 128(42.4%) were housewives. Regarding to age distribution, the mean and standard deviation of participant’s age was 45.57 (SD = 13.77) (Table 1).

**Table 1:** Baseline characteristics of cancer patients in UoGCSH, and FHRH 2019.

Variables	Frequency	Percentage
**Sex**		
Male	108	35.8
Female	194	64.2
**Marital status**		
Single	27	8.9
Married	221	73.2
Divorced	26	8.6
Widowed	28	9.3
**Educational level**		
No education	150	49.7
Primary education	43	14.2
Secondary education	63	20.9
College and above	46	15.2
**Occupation**		
Government employee	73	24.2
Farmer	50	16.6
Merchant	35	11.6
Unemployed	144	47.68
**Monthly Income (ETB)**		
< 1000	47	15.6
1000–1999	103	34.1
2000–2999	63	20.9
> 3000	89	29.5

### Behavioral and co-morbidity characteristics

Four participants (1.3%) were declared that they were smoking cigarettes daily and 42 (13.9%) were alcohol consumers. Most of the participants 226(74.8%) were physically active. Most of the participants 200(66.2%) were in normal weight category based on their BMI whereas 63(20.9%) and 39(12.9%) were underweight and overweight respectively. Ninety-two participants (30.5%) had additional co-morbidity (Table 2).

**Table 2:** Behavioral factors and co-morbidities among study participant at UoGCSH, and FHRH 2019.

Variables	Frequency	Percentage
Smoking status		
No	298	98.7
Yes	4	1.3
Alcohol drinking		
No	260	13.9
Yes	42	861
Physical activity		
No	76	25.2
Yes	226	74.8
Body mass index		
< 18.5	63	20.9
18.5–24.9	200	66.2
>25	39	12.9
Co-morbidity		
No	210	69.5
Yes	92	30.5
Diabetes		
No	279	92.4
Yes	23	7.6
Hypertension		
No	274	90.7
Yes	28	9.3
Anemia		
No	273	90.4
Yes	29	9.6
HIV/AIDS		
No	292	96.7
Yes	10	3.3

### Type of cancer and treatment-related characteristics

Breast cancer was the commonest 79(26.2%) cancer. Whereas cervical cancer, 37(12.3%), colorectal cancer35 (11.6%) and lung cancer 34 (11.3%) are ranked second to fourth. Most of the patients, 164(54.3%), were diagnosed with the disease in the past six months prior to the study. Regarding the clinical stage of the disease, the third stage accounts for 90(29.8%) patients. A total of 130 (43%) participants have taken chemotherapy for less than three months (Table 3).

**Table 3:** Type of cancer and treatment-related characteristics of study participant at UoGCSH, and FHRH 2019.

Variables	Frequency	Percentage
**Type of cancer**		
Breast cancer	79	26.2
Lung cancer	34	11.3
Colorectal cancer	35	11.6
Gastric cancer	10	3.3
Cervical cancer	37	12.3
Head and Neck cancer	22	7.3
Esophageal cancer	10	3.3
Blood cancer	11	3.6
Skin cancer	4	1.3
Thyroid cancer	3	1.0
Bladder cancer	14	4.6
Lymphoma	18	6
Liver cancer	14	4.6
Sarcoma	5	1.7
Testicular cancer	6	2.0
**Clinical stage**		
Stage 1	29	9.6
Stage 2	86	28.5
Stage 3	90	29.8
Stage 4	62	20.5
Unknown	35	11.6
**Duration since diagnosis**		
< 6 months	164	54.3
7–12 months	98	32.5
> 12 months	40	13.2
**Duration since start of chemotherapy**		
1–3 months	130	43
4–6 months	100	33.1
>6 months	72	23.8

### Prevalence of depression among cancer patients

**Fig 1:**
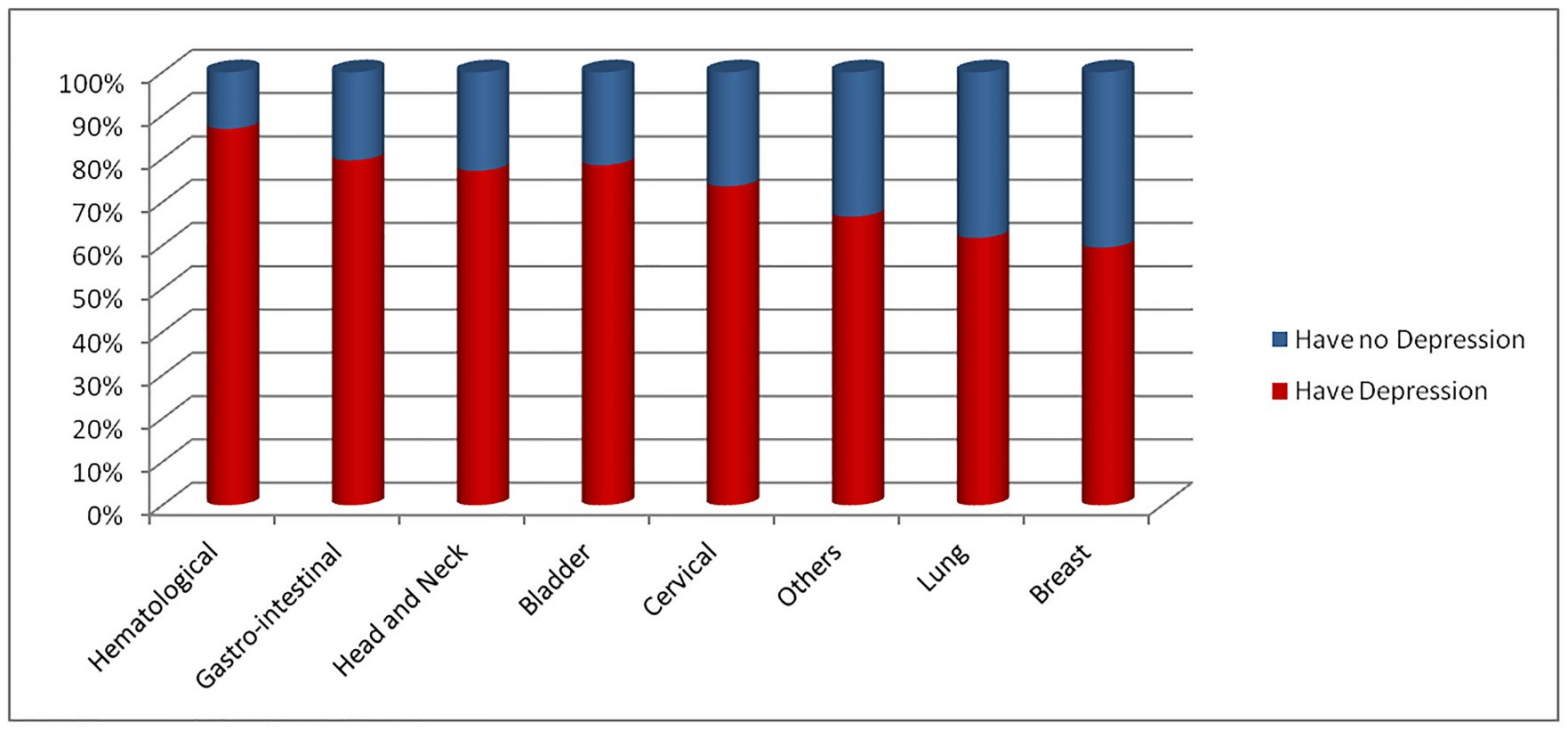
Prevalence of depression among cancer patients on chemotherapy in UoGCSH and FHRH, northwest Ethiopia.

In this study 214 patients had depression making the prevalence 70.86% (95% CI: 65.38, 75.92). The prevalence of depression among male cancer patients was 77.78% (95% CI: 68.76, 85.21) whereas it was 67.01% (95% CI: 59.91, 73.58) among female patients. From the total patients with depression, 146 (68.2%), 49 (22.9%), and 19 (8.9%) had moderate, moderately severe, and severe depression respectively. The magnitude of depression has also shown the difference among different types of cancer (Fig 1).

### Factors associated with depression among cancer patients

In bi-variable logistic regression age, sex, marital status, educational status, occupation, BMI, Social support and duration of chemotherapy were found to have P-value < 0.2subsequently these variables were subjected to multivariable analysis and educational level, occupational status BMI status and duration of chemotherapy were statistically associated with depression among cancer patients.

The odds of depression among patients who attended college and above was significantly reduced when compared to those with no education (AOR = 0.1, 95% CI: 0.02, 0.43). When compared to government employees patients who are unemployed had less risk of depression (AOR = 0.15, 95% CI: 0.04, 0.58). Underweight patients had 2.39(AOR = 2.39, 95% CI: 1.10, 5.19) times higher odds of depression as compared to those with normal body mass index. Patients who took chemotherapy for six months or more had 2.36 (AOR = 2.36, 95% CI: 1.16, 4.79) times higher odds of depression as compared to their counterparts (Table 4).

**Table 4:** Factors affecting depression among cancer patientsat UoGCSH, and FHRH 2019.

Variables	Depression		COR (95% CI)	AOR(95% CI)
	Yes	No		
Age (mean, sd)	46.4(13.3)	43.5(14.8)	1.02 (0.99, 1.04)	1.01(0.98, 1.03)
**Sex**				
Male	84	24	1	1
Female	64	130	0.58 (0.34, 0.99)	0.6 (0.26, 1.40)
**Marital status**				
Single	16	11	1	1
Married	162	59	1.89 (0.83, 0.43)	1.14 (0.38, 3.46)
Divorced	16	10	1.10 (0.37, 3.31)	0.69 (0.18, 2.68)
Widowed	20	8	1.72 (0.56, 5.28)	1.0 (0.21, 4.57)
**Educational level**				
No education	109	41	1	1
Primary	34	9	1.42 (0.63, 3.22)	1.02 (0.40, 2.61)
Secondary	45	18	0.94 (0.49, 1.81)	0.42 (0.15, 1.18)
College **+**	26	20	0.49 (0.25, 0.97)	0.1 (0.02, 0.43)*
**Occupation**				
Government	52	21	1	1
Farmer	40	10	1.62 (0.68, 3.81)	1.5 (0.35, 1.78)
Merchant	27	8	1.36 (0.53, 3.48)	0.30 (0.07, 1.28)
Unemployed	95	49	0.78 (0.42, 1.45)	0.15 (0.04, 0.58)*
**Body mass index**				
Underweight	52	11	2.17 (1.06, 4.45)	2.39 (1.10, 5.19)*
Normal	137	63	1	1
Over weight **+**	25	14	0.82 (0.4, 1.69)	0.86 (0.38, 1.95)
**Social support**				
Poor	62	21	1	1
Moderate	90	34	0.9 (0.48, 1.69)	0.97 (0.49, 1.92)
Strong	62	33	0.64 (0.33, 1.22)	0.62 (0.31, 1.27)
**Duration of chemotherapy**				
≤6 months	155	75		
> 6 months	59	13	2.2 (1.13, 4.25)	2.36 (1.16, 4.79)*

* P-value < 0.05.

## Discussion

In this study, we have assessed the magnitude of depression among cancer patients and the factors affecting it. We have found 70.89% prevalence and occupation, educational status, body mass index and duration of chemotherapy were found to be independent predictors of depression.

The magnitude of depression in this study was consistent with other studies conducted among Chinese cancer patients (66.72%) [8] whereas this figure was higher than a study conducted in Addis Ababa [12], Iran [4] and meta-analysis done by Krebber et al. [7]. This discrepancy could be attributable to the difference in the study populations in terms of types of cancer, the tool used for screening or other socio-demographic variations and severity of depression considered.

The odds of depression was significantly reduced in patients who are unemployed when compared to government employees. This piece of evidence is supported by another multicenter study [11]. This could be related to work-related stress which worsens feelings of inadequate control over one’s work, frustrated hopes and expectations leading to depression [24].

The odds of depression among patient who attended college and above was reduced when compared to those who have no education. This finding is supported by a study from China [8], Atlanta [25] and Greece [26]. The possible reason could be these patients may have a better understanding of the disease and have early screening which increases their recovery. A higher proportion of educated people (36.96%) are in the first or second clinical stage of cancer as compared to 25.3% of patients without education.

Underweight cancer patients had more than double odds of depression as compared to those who have a normal body mass index. This finding is supported by several single studies [27, 28], and systematic review and meta-analysis [29] showing underweight people at higher risk of depression. This shows malnutrition has a significant role in the mental health of people and maintaining a healthy weight is essential to improve health in general and mental health in particular.

Even though the chemotherapy duration has shown no significant association with depression in few pieces of literature [30, 31]. The odds of depression among cancer patients who took chemotherapy for more than six months was higher than their counterparts in this study. This could be a side effect of chemotherapy [32] or it could be also associated with the staggering cost of chemotherapy which makes these patients stress to buy it for an extended duration.

This study assessed the frequently ignored aspect of cancer co-morbidity, depression. But the study has some limitations as it was a cross-sectional study. The cause-effect relationships are not guaranteed in these studies; therefore we recommend a prospective study. Even though we have used a validated tool, some of the symptoms used in PHQ– 9 like weight loss and tiredness might be related to cancer itself and may overestimate depression.

## Conclusion

The burden of depression among cancer patients in this study was high. Occupation, educational status, body mass index and duration of chemotherapy were found to be independently associated to depression. We recommend concerned bodies working to curve the problem to intervene based on the identified risk factors. Improving educational status, reducing work stress and maintaining normal weight would reduce depression. Clinicians shall also provide integrated care of mental health and cancer treatment.

## Abbreviations

AOR: Adjusted Odds Ratio
CI: Crude Odds Ratio
COR: Crude Odds Ratio
PHQ: Patient Health Questionnaire
SD: Standard Deviation
UoGCSH: University of Gondar Comprehensive Specialized Hospital
